# Nature Archetypes – Concepts Related to Objects and Phenomena in Natural Environments. A Swedish Case

**DOI:** 10.3389/fpsyg.2020.612672

**Published:** 2021-01-14

**Authors:** Johan Ottosson, Patrik Grahn

**Affiliations:** The Department of People and Society, Business Economics and Environmental Psychology, Swedish University of Agricultural Sciences, Alnarp, Sweden

**Keywords:** health-promoting properties in nature, properties of natural environments, health promotion, perception, characteristics, stress, calm and connection system

## Abstract

Do people classify nature in ways that can be described as archetypes? Could it be that these can be interpreted as health promotive? More and more researchers today suggest that archetypes can be used to analyze, describe, and develop green spaces. In parallel, an increasing number of research results since the 1980s have shown that human health and well-being are positively affected by stays in certain nature areas. The qualities in these nature areas which stand out to be most health-promoting are interpreted to be properties of nature that humans through evolution are prepared to perceive in a positive way. In this study, 547 respondents in southern Sweden answered a questionnaire on properties in natural areas. Through cluster analysis, these properties have been grouped into 10 types of nature and landscape. The 10 clusters are related to specific phenomena and places in Scandinavian nature, which could be described as archetypal. These natural phenomena and places are discussed, partly based on references to archaic Scandinavian mythology, Scandinavian lifestyle, and cultural canon, and partly on research on evolution, human preferences, and how nature can affect human health. We discuss how these nature archetypes evoke anxiety, fear, and distancing as well as calmness, tranquility, and connection. However, researchers have so far focused on how visits to natural environments have affected the sympathetic nervous system, and not realized the possibility of including the calm and connection system as well as the oxytocin in their explanatory models. In a follow-up article, we intend to develop a model for how the nature archetypes can interact with the calm and connection system.

## Introduction

Since time immemorial, we humans have had an interest in seeking to explain the great existential story and in placing ourselves and our activities in the universe, often through religious beliefs (Turner, 2005). Various parts of the natural landscape have thus been described as more friendly and divine than others. Examples can be taken from many cultures, such as the Arcadia of the ancient Greeks, the Paradise of the Persians, and the Eden of the Bible. In descriptions of these places, we find trees with good, edible fruits, plenty of fresh water, and friendly animals (Prest, 1988; Gerlach-Spriggs et al., 1998). In the Bible’s Creation, we meet Adam and Eve in the Garden of Eden. In Ancient Greece, Arcadia is described as a place of happiness and beauteous perfection. The Epic of Gilgamesh describes a garden of gods, a paradise as a place where even sicknesses give way and where we may regain power and strength (Stigsdotter, 2005). None of these environments emphasizes buildings – it is nature and the gardens that are highlighted. But, nature also contains dangerous places, animals, and phenomena, which in various religions (e.g., Christianity and Islam) are tied to evil demons or the devil, and where the malefic underworld (e.g., Hades or Hell) is illustrated as a dark place, with forests where one gets lost; deserts and swamps, and being extremely hot or cold. This may best be seen in Dante’s Divina Comedia, with amazing illustrations of the underworld by Gustave Doré (Alighieri, 2018).

These archaic descriptions of the essence of different natural landscapes can be described as nature or landscape archetypes. Today, holistic, easily understandable, and relevant descriptions of natural areas are needed: relevant, not least when it comes to the importance of nature areas for human health and well-being. Bourassa (1988) proposed a theory to be used in landscape architecture and planning: When analyzing landscapes, he argued, it may be appropriate to use Jung’s concept of archetypes. According to Bourassa (1988), modern environmental psychological theories can be interpreted as being in line with CG Jung’s thoughts on archetypes. Just under a decade later, Nash (1997) suggested that CG Jung’s concept of archetypes could be used to understand how landscapes have been perceived and interpreted throughout history. Today, the term archetypes is used increasingly in scientific publications when landscapes are to be described, such as Hreško et al. (2015), Wilkinson (2015), Wardropper et al. (2016), Cullum et al. (2017), Jin and Du (2017), Catalani et al. (2018), Hartel et al. (2018), Nogué and Wilbrand (2018), Olszewska et al. (2018), Xing and Chen (2018), and Evers et al. (2019). Several of the researchers claim that in landscape planning and landscape architecture, it is necessary to capture and describe a holistic meaning in the landscape and suggest that archetypes are the solution. Hreško et al. (2015) claim that: “Several contemporary works of landscape ecologists deal with the problem of landscape type determination with the emphasis on the synergy of multidimensional landscape perception.” The authors mention the physical part of the landscape and its characteristics, as well as the evaluation of landscape changes relating to landscape diversity and the perception of “cultural-spiritual entity.” Cullum et al. (2017) suggest the following: “We propose the use of archetypes as a way of moving between conceptual framings, empirical observations, and the dichotomous classification rules upon which maps are based. An archetype is a conceptualization of an entire category or class of objects. Archetypes can be framed as abstract exemplars of classes, conceptual models linking form and process and/or tacit mental models.” The link to CG Jung’s theories of archetypes is shallow in these two articles (Hreško et al., 2015; Cullum et al., 2017). In other articles, the link to Jung’s theories is much more prominent: Wilkinson (2015) wishes to create more meaningful landscape architecture through an increased understanding of the complex relationship between people and nature. One way to increase this knowledge is through the use of Jung’s theories of archetypes, she suggests. When Olszewska et al. (2018) formulate categories regarding what makes certain landscapes be experienced as contemplative, they refer to Jung (1964), and this applies in particular to their proposed category of “archetypal elements,” such as stones, an old tree, or a path. Xing and Chen (2018) argue, with reference to CG Jung, that “the design of a public space should attach importance to the change in people’s emotional experience according to their surroundings. Emotion comes from human’s collective unconsciousness; whose content is archetype.”

### Nature and Landscape Archetypes

Bourassa (1988), Nash (1997), Wilkinson (2015), and Xing and Chen (2018) all see great opportunities to be able to develop both theory and practice in landscape architecture and landscape planning through Jung’s theory of archetypes. The origins of the archetypal hypothesis date back to ancient Greek civilization and Plato. Plato’s “Eidos” were pure mental forms that were imprinted in the soul before the person was born into the world (Williamson, 1985). They were collective in the sense that they embodied the fundamental characteristics of a phenomenon rather than its specific particularities. Philo of Alexandria developed the thoughts about Eidos and began to use the term archetypes (Hillar, 1998). The concept of psychological archetypes we know today was primarily elaborated by Jung (1968). In his psychological framework, archetypes are inherent, universal prototypes for ideas. An example above is how Olszewska et al. (2018) propose a category of “archetypal elements,” e.g., stones, an old tree, or a path. CG Jung, however, does not just write about archetypal elements. Jung’s theory of archetypes can be seen as a number of spherical layers (Sharp, 1991). The layer that lies outermost is conscious; the next layer is the personal unconscious with a number of unconscious structures or complexes. The content of this layer is unique to each person, and the structures you have developed depend on what predispositions you have, what you have experienced, and how you have managed your experiences. The next layer is the collectively unaware, with a variety of inherited archetypes. Examples are symbolic archetypes or elements, such as snake, tree, sun, moon, and fire (Jung, 1964, 1968; Sharp, 1991). In addition, there are character archetypes, such as the parent, the hero or god; as well as situational archetypes or experiences, such as birth, death, marriage, and lost love. Jung considered archetypes as something evolved through evolutionary processes. The archetypes affect how a person experiences and interprets the phenomena that he or she observes. They can induce experiences and feelings such as fear and happiness that can be related to key archetypes such as the parent, hero, or god. The subconscious makes itself known through symbols found throughout life: in dreams, art, and religion and moreover in human behavior patterns and relationships (Jung, 1964, 1968; Sharp, 1991). CG Jung argued that similarities between legends/myths from different cultures reveal the existence of universal archetypes (Boeree, 2017).

Jung expressed the connection of archetypes and the collective unconscious to evolution as that they are based on “the deposits of all our ancestral experiences, but they are not the experiences themselves” (Jung, 1928). How should this be understood? First, several researchers (Hogenson, 2001; MacLennan, 2006; Stevens, 2012; Oatley, 2019) claim that Jung was strongly influenced by Baldwin (1896a,b): Baldwin’s theory is about the ability of a species’ individuals to learn to handle specific situations, enabling the species to survive. This creates the conditions for shaping selection processes until the genetic composition of the species is developed to be able to handle the environment reflexively and independently. Behavioral flexibility can, according to Baldwin (ibid.), solve the adaptive problem between environment and individual faster and more successfully than through purely random selection. Evolution can thereby, in humans, be driven forward by cultural influence. Hogenson (2001) exemplifies this with the fact that animal husbandry with cows eventually led to more and more people being able to tolerate lactose better. These mechanisms have been going on for millions of years, and have affected human relationships and behavior in natural environments as well as in social contexts. Second, Bergson (2007) argued that processes based on immediate experience and intuition are more important for understanding reality than abstract rationalism and science. On the basis of Bergson, Jung proposed that human beings in addition to instinct uses intuition, which Jung claimed enables the premonition of situations in which human’s perception of complex situations contributes to the development of the human being (Jung, 1960, p. 269). Jung argued that the combination of this type of intuition: “namely, the archetypes of perception and apprehension” with instincts constitutes the collective unconscious (Jung, 1960, p. 270). Partly based on Bergson and Baldwin, Jung argues that this premonition and collective unconsciousness develops over time and is incorporated into people as a type of cultural learning, intuitive understanding, and flexibility in behavior. Man’s relationship to nature and archetypal references to nature, through myths, legends, and instincts, must be understood against this background.

Jung’s theory was that some strong archetypes have been found in all cultures, since time immemorial. Natural environments have followed human beings through evolution, so in that sense there should be strong archetypes related to both objects and events in nature. Nevertheless, Jung did not propose any specific nature archetype, despite the fact that many myths and legends are linked to particular types of natural areas. These are also associated with mythological figures. However, it can be interpreted as it was on the verge that Jung proposed habitat types. During his travels in Africa, Mexico, etc., Jung met many representatives from indigenous people and wrote on the basis of these experiences about the archaic man. An archaic person does not believe in god, he knows that god exists, Jung said (Sabini, 2008). This is not least because god is tangible, you can experience god purely physically; see, hear or feel god. Archaic religions are usually intimately associated with natural phenomena such as the sun, the moon, thunderstorms, etc. However, Jung may have suspected that it might be considered a blasphemy to call these phenomena, linked to gods, archetypes. In Sabini (2008, p. 114), Jung describes an incident where a Pueblo chieftain points to the sun and calls it his god, his father. C. G. Jung was very interested in nature and loved being there. He also claimed that human beings have a need to be in natural environments in order to stay whole and healthy. Especially for our mental health, he argued, it is important to have contact with nature: “Natural life is the nourishing soil of the soul” he said, and: “Human existence should be rooted in the earth” and: “Nature is an incomparable guide if you know how to follow her” (Sabini, 2008).

Today, more and more researchers are studying theories regarding archetypes in different scientific disciplines (Winborn, 2016). Among others, Hogenson (2001), MacLennan (2006), Stevens (2012), and Oatley (2019) argue that modern research shows that archetypes and the collective unconsciousness can be linked to evolution, and also to epigenetics. In cross-cultural empirical studies, research results seem to support that people have an inherited ability to recognize symbols and understand their meaning (Sotirova-Kohli et al., 2013). Researchers also discuss the relationship between archetypes and new research findings on epigenetics (Anderson, 2016; Roesler 2018). In this article, we will use the concept of nature archetypes. In ancient Greek philosophy, arche signifies the origin or the principle of a thing. Typos is an ancient Greek word which means model, impression, or form.

### Environmental Psychological Theories and Findings Which Could Be Linked to Archetypes

The booming interest in Jung and archetypes shown by researchers in landscape architecture and landscape planning, may possibly be explained in part by the fact that rapid globalization and urbanization is making public urban green spaces throughout the world increasingly uniform, but perhaps mainly because more and more research shows that people’s stay in nature affects their health in a positive way. Studies show that nature and gardens do affect human health positively, especially when it comes to reducing stress levels and rehabilitating people affected by stress-related mental illness (Egorov et al., 2016; Grahn et al., 2017; van den Bosch et al., 2018; Nilsson et al., 2019). However, we know too little about what qualities natural areas should have, in order to be health promoting. Most theories that seek to explain health effects are based on evolutionary hypotheses; that is, human beings’ roots or ancient “home” are to be found in certain natural landscapes. Examples are the Attention Restoration Theory (Kaplan, 2001), the Stress Reduction Theory (Ulrich, 1993), the Supportive Environment Theory (Grahn et al., 2010; Stigsdotter et al., 2011; Adevi, 2012), and the Biophilia Hypothesis (Wilson, 1984).

Fromm (1964) used the term “biophilia” to describe a mental, emotional/instinctive, attraction to all that is alive and vital. The biophilia hypothesis, later developed by Wilson (1984), proposes that the human being has an innate tendency to focus on life-forms in natural landscapes. He claims that it is an “urge to affiliate with other forms of life” or “the connections that human beings subconsciously seek with the rest of life.” The strongest expressions of the biophilia hypothesis are its opposite: biophobia (Ulrich, 1993). Human beings react extremely quickly and strongly to biophobic phenomena like snakes, spiders, darkness, steep cliffs, and blood (Ulrich, 1993; Ottosson et al., 2015). There are some common features of the natural elements that these theories propose as health promotive. As far back as the 1970s, Appleton (1975) suggested that we probably have an innate preference for what are known as prospect/refuge locations in nature, such as forest edges. Orians (1980, 1986) and Orians and Heerwagen (1992) suggested that through evolution human beings have developed a high preference for savannah-like environments: grassy areas where trees grow sparsely; it is moderate with shade and the ground is usually reached by the sunlight. In such environments, Orians (1986) claims that human beings appeared as a species millions of years ago. Balling and Falk (Balling and Falk, 1982; Falk and Balling, 2010) suggested that human beings have a special innate love for trees with wide canopies, which are also found on the savannah. The environmental qualities described by Appleton (1975, 1990), Balling and Falk (1982), and Orians (1986) are proposed by Ulrich (1993) to reduce stress. He asserts that natural environments should contain a depth, i.e., too open natural environments such as deserts and too dense natural environments create insecurity and thus do not reduce stress, promote restoration and health promotion (Ulrich, 1993).

## Aims and Objectives

The hypothesis is that people, to a significant extent, categorize objects and phenomena in natural environments based on inherited mechanisms. We assume that there is a connection between different objects/phenomena in nature, based on their associations with inherited impulses and behaviors. Archetypes – symbols, characters, and situations – are fundamental elements of religions, myths, legends, and folk tales (Sharp, 1991). Our intention is, therefore, to describe such contexts, from archaic time till today, based on connections in our empirical material. The purpose of the study is to investigate whether there is a clear connection between nature experiences, and whether these relationships form separate entities that can be described and interpreted based on theories related to archetypes and/or biophilia. The analysis aims to summarize a large variety of natural elements/natural phenomena to a manageable level and illustrate their relationship. Our hypothesis is that these relationships can be explained based on people’s more or less subconscious positions. These relationships can possibly be used to better understand how human beings are influenced by nature.

In addition, we assume that these clusters of natural phenomena evoke similar experiences in most individuals. Emotions linked to such archetypal experiences should be linked to basic emotions, such as fear, avoidance, and stress or to positive feelings of wellbeing, relaxation, and feelings of safety.

## Materials and Methods

In order to understand how people categorize objects and phenomena in nature, it was decided that an extensive questionnaire should be created. Nature can be defined as the phenomena of the physical world collectively, including plants, animals, the landscape, and other features and products of the earth (Oxford English Living Dictionaries, 2019). According to Johnson et al. (1997), the natural environment consists of all living and non-living things occurring naturally. It encompasses the interaction of all living species, weather, climate, and natural resources that affect human survival, including animals, vegetation, soil, rocks, atmosphere, and natural phenomena that occur within their boundaries and their nature.

This study was delimited to southern Sweden, with its characteristic type of natural landscape. Southern Sweden’s nature belongs to the nemoral natural geographic region, representing the main part of Central Europe, with forests of deciduous trees like European beech (*Fagus sylvatica*), European ash (*Fraxinus excelsior*), hornbeam (*Carpinus betulus*), and a limited presence of coniferous forests, mainly Norway spruce (*Picea abies*) and Scots pine (*Pinus sylvestris*; Paine, 2008). Closer descriptions of characteristics of the southern Swedish landscape are depicted in larger compilations, such as the National Atlas of Sweden (Nordiska ministerrådet, 1984; Selander, 1987; Helmfrid et al., 1994; Raab and Vedin, 1995).

Based on the above, a comprehensive questionnaire was constructed by the authors, where a large number of objects and phenomena in natural environments in southern Sweden were listed. These were intended to include as many types of significant elements as possible, in terms of experiences in natural environments that people can relate to. These elements include vegetation, animals, and landscape formations such as mountains, lakes, and watercourses as well as the cultural elements that usually belong here, such as roads, paths, buildings, and enclosures. Common weather types and celestial phenomena are also included. The most recognizable and relevant elements for the public were included in the questionnaire. This list was sent to eight colleagues in environmental psychology and landscape architecture, who included suggestions for additions. The final list contained 261 objects and phenomena in the natural environment in Southern Sweden. The aim was for collected data to be analyzed using multivariate statistical programs, such as cluster analysis.

Cluster analysis groups a set of objects in such a way that objects in the same group – a cluster – are more similar in some sense or another to each other than to objects in other clusters. Results from cluster analyses must be interpreted in order to become meaningful (Everitt, 1980; Rokach and Maimon, 2005). There are many different types of cluster analyses. Hierarchical clustering, which we chose to use, connects objects to form clusters based on their distance. This can be represented using a dendrogram, which explains the name “hierarchical clustering”: these algorithms provide an extensive hierarchy of clusters that merge with each other at certain distances. In a dendrogram, the *y*-axis marks the distance at which the clusters merge, while the objects are placed along the *x*-axis such that the clusters do not mix.

The comprehensive questionnaire was initiated with a tutorial: “Do not think too long about what to answer. Your spontaneous response is usually the correct one.”

Then the question:

“How much do you like ….”

The response options were from 1 = not at all to 5 = a lot. There were opportunities to add more things – such as specific types of rocks, lakes, sounds, or smells. Some took advantage of this opportunity but later discovered that these questions came further on in the form. Therefore, we received no unique additions to the questionnaire from the respondents.

The form contained no pictures, only linguistic formulations, which means that individuals themselves were allowed to form their own opinion. Presenting an image limits the imagination of the respondents. For example, we wrote “small country lane with green in the middle,” or “pick-stone,” where the person could refer to their own memories of small stones and roads – and how they can be further linked to environments with trees, herbs, and landscape formations; associations that ultimately form clusters. The objects in the questionnaire were grouped with, for example, trees, smells, and sounds separately.

The questionnaire was sent to respondents 1 or 2 weeks before they attended a lecture. They were required to fill out the questionnaire before attending. The purpose of the lectures was to extract a number of fully completed questionnaires. The lecture in itself was a standard lecture in the regular course provision. In total, there were 19 lectures for nursing staff in their further education programs (124 respondents), students of medicine or nursing in the regular course provision (278 respondents), and patients and or people living in nursing homes in their regular range of activities and lectures (145 respondents). Overall, there were 547 respondents, of whom 454 were female and 92 were male (1 missing data). Sixty respondents were foreign-born but most had grown up in or lived in Sweden for a long time. None of the respondents had any difficulties in interpreting and filling in the questionnaire, which was in Swedish. Thirty-one respondents were born in the Nordic countries, 22 in the rest of Europe, and 7 in countries outside Europe. Of those born in the Nordic countries, most came from Finland (17), followed by Denmark (9) and Norway (5). Of the informants who came from the rest of Europe, most came from Poland (9) and the former Yugoslavia (7).

The Ethics Committee at Lund University Approved the Study. Connectivity based clustering is a group of methods that differ in the way distances are computed. We used Ward’s cluster method to group the answers (Ward, 1963). Ward’s method is suitable to use when dealing with quantitative variables with multiple response options. Ward’s minimum variance criterion minimizes the total within-cluster variance. Hence, Ward’s method provides clear clusters, well separated from each other. Input data from the questionnaire (Likert items) were ordinal data. We used SAS Proc Distance, Method = Euclid, to translate ordinal data to Euclidian distances. The new data set was put into Proc Cluster Method = Ward. Wards minimum-variance cluster method uses squared Euclidian distance data. This method is based on agglomerative hierarchical clustering procedure: each variable begins as a cluster by itself. The two closest clusters are then merged to form a new cluster that replaces the two old clusters. Merging of the closest clusters is repeated until one single cluster is left. A shortcoming in the method is that it is sensitive to outliers (Milligan, 1980). For this reason, we chose to use SAS TRIM option, and, in addition, we used the SAS method Centroid to define outliers. The centroid method is more robust to outliers than most other hierarchical methods but in other respects, does not perform as well as Ward’s method (SAS Institute Inc, 2018).

**Figure 1:**
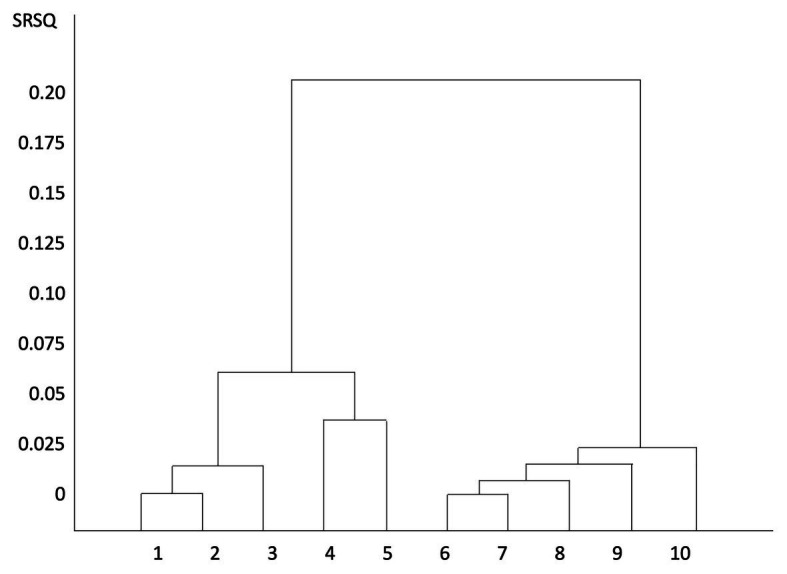
Cluster dendrogram. The dendrogram is based on the participants’ preferences for different qualities in nature. *Y*-axis, semipartial R-Square; *X*-axis, clustered preferences.

The semipartial R-square (SRSQ) is a measure of the homogeneity of merged clusters. That is, how similar the cluster elements are to each other. The SRSQ value should be as small as possible to imply that two homogeneous groups should merge. The SRSQ values are used when drawing dendrograms of how the clusters relate hierarchically to each other, their relationship. Very small values of SRSQ imply that these are not plotted in the dendrogram (see Figure 1). The dendrogram is helpful in determining how many clusters are relevant to consider as independent. In addition to the visual assessment using the dendrogram, some statistics are available for determining the number of clusters. Three types of statistics are considered: First, the root-mean-square standard deviation (RMSSTD), which is a measure of homogeneity within clusters. A marked decrease or increase of RMSSTD-values may indicate that a satisfactory number of clusters have been reached. Second, we used Pseudo F statistics (PSF) as guidance for finding the appropriate number of clusters. If PSF receives a clearly higher value, cluster analysis has a stability, which means a proposal for the best number of clearly separated clusters. Third, we used Pseudo T2 statistics (PST2). Where PST2 have a clearly lower value, the analysis is more stable. The ideal is where PST2 has a lower value where PSF also has a higher value and that a decrease or increase in RMSSTD values is also found, which together indicates that a satisfactory number of clusters have been achieved (Milligan and Cooper, 1985; Bruun Brockhoff et al., 2005; SAS Institute Inc, 2013, 2018). SAS Software 9.4 was used in the statistical analyses.

As already mentioned, results from cluster analyses must be interpreted to become meaningful (Everitt, 1980; Rokach and Maimon, 2005), and our ambition was to describe archetypal wholes. In order to understand how people connect different objects and phenomena in natural areas to special entities, these must be put into their cultural context.

The results were interpreted based on references to research in, for example, environmental psychology and landscape architecture. Our aim was to investigate whether it was possible to interpret the clusters based on archaic conceptions, linked to southern Sweden. The participants in the study are Swedish, as are the researchers in the study. We, therefore, chose to interpret the connections based on a Western cultural and literary canon (Bloom, 1994), and in particular based on a Swedish cultural and literary canon. It includes the Bible as well as myths, fairy tales, and gods that are linked to Swedish pre-Christian religion. These beliefs belong to world of the ancient gods, with heroic fighters – the Æsir gods, and those bringing fertility – the Vanir gods. There are also elves, trolls, and creatures that belong to Scandinavian folklore. The literary canon includes novelists such as Selma Lagerlöf and Harry Martinson, poets such as Tomas Tranströmer, composers such as Evert Taube, children’s book authors such as Astrid Lindgren, filmmakers like Ingmar Bergman, and painters such as John Bauer and Anders Zorn; and in addition, books, movies, TV series, streaming media, etc., from the present day.

The lists of variables were read many times, and the members in the research group put in every effort to evoke comprehensive interpretations that could serve as nature archetypes (Jung, 1964, 1968) and/or holistic Gestalts (Perls et al., 1951).

## Results

The SAS TRIM option and the Centroid analysis suggested that four variables were outliers: “cloudberries,” “the buzz of the mosquitos,” “mixed conifer forests,” and “waterfalls.” The participants in the study came in most cases from southern Sweden where these phenomena are unusual, which might explain why they became outliers. These variables, therefore, came to be removed from the analysis, which thus contained 257 variables.

Table 1 presents the results of the cluster analysis. The cluster analysis starts with 257 variables, which are merged step by step to finally become a single cluster. Table 1 shows the last 15 steps. The left column shows the number of merged clusters, column two shows which clusters are merged and column three, semi-partial R-square, shows the distance between the clusters. Column four, RMSSTD, presents peaks at 13, 10, and 5 clusters. Column five, PSF, does not show any particular plateau of high values that can assist in the analysis. Column six, PST2, has a first clear low point at 11 clusters and a second low point at 13 clusters. The appropriate number of clusters should not be too high. Therefore, 10 or 11 clusters would be more appropriate than 13. The final choice of clusters is determined after a more qualitative evaluation. The final result came to be 10 clusters, because clusters 10 and 11 lay very close to each other in the cluster tree and were easier to interpret as one cluster instead of two (see cluster 5 in Table 2).

**Table 1:** SAS Ward’s cluster analysis of the respondents’ responses to the questionnaire.

Number of clusters	Merged clusters	SRSQ	RMSSTD	PSF	PST2
15.	Cl22-Cl30	0.0108	0.0738	24.2	7.0
14.	Cl27-Cl29	0.0116	0.0725	24.9	8.0
13.	Cl16-Cl20	0.0120	0.0870	25.8	5.2
12.	Cl25-Cl113	0.0122	0.0694	26.9	9.5
11.	Cl33-Cl13	0.0131	0.0880	28.2	5.5
10.	Cl31-Cl60	0.0170	0.0941	29.4	8.6
9.	Cl19-Cl17	0.0199	0.0844	30.7	10.2
8.	Cl14-Cl26	0.0202	0.0752	32.5	12.9
7.	Cl12-Cl8	0.0227	0.0777	34.7	12.7
6.	Cl7-Cl21	0.0274	0.0796	37.4	14.5
5.	Cl9-Cl53	0.0334	0.0919	40.8	15.0
4.	Cl6-Cl15	0.0381	0.0830	46.4	18.5
3.	Cl10-Cl11	0.0485	0.1001	56.0	18.1
2.	Cl5-Cl3	0.0744	0.1055	76.5	23.7
1.	Cl2-Cl4	0.2267	0.1066		76.5

*N* = 546.

**Table 2:** Variables regarding objects and phenomena in the natural environment in southern Sweden that are included in Cluster 5.

Variable number	Object
168	The croft/cottage
169	The stable and byre
170	The barn
167	The farm
198	Farm animals like chickens, sheep, horse, and cow
50	The sky
60	The horizon
55	The starry sky
56	The moonlight
57	The full moon
58	The crescent
59	The northern lights
78	Snowfall
237	The scent after snowfall
79	The snowy landscape
80	Snow on trees and branches
263	Complete silence
65	Small clouds
191	The verge of the road
21	Stones – less than a fist
22	Small picked stones
209	The ditch
23	The bare ground
204	The allotment
88	The young tree
33	The young forest
200	The park
165	The cemetery
136	The pigeon
150	The swan
31	The deep forest
32	The old-growth forest
33	The primeval forest
34	The mountain forest
25	The alpine mountains, the fjeld
37	The coniferous forest
39	The pine forest
38	The spruce forest
99	The pine
100	The spruce
113	Berries
114	Blueberry
115	Lingonberry
116	Wild strawberries
118	Mushrooms
119	Chanterelle
8	The forest lake
7	The big lake
9	The flatland lake
62	Fish
63	Snail

Tables 2–11 show which variables are included in the 10 clusters. We have chosen to include the numbers from the questionnaire. From this, it can be seen that the clusters have variables from several sections in the questionnaire.

Figure 1 shows a dendrogram over the cluster analysis. To begin with, it shows two very distinctly spaced main branches, with five clusters in each. The left main branch is divided into two distinct parts, with three clusters to the left and two to the right. The right main branch divides further down, suggesting that the clusters contained here are not as clearly separated. The right main branch divides with one cluster to the right and four clusters to the left.

The 257 different phenomena in nature came to be delimited into 10 separate clusters in the analysis. The result is interpreted below with references to archaic Scandinavian mythology, Swedish art, literature, and Christian religion, and to other research. To experience a landscape, with all its natural vicissitudes during the various phases of the day and the year, includes the whole body, all the senses. The landscape becomes a part of us, just as we become a part of it (Scriven, 2014). The landscape is hence perceived as situated and embodied (Ladouce et al., 2017; Adevi et al., 2018), and the discussion is based on this.

### The Demanding Landscape

The dendrogram of the cluster analysis is divided into two distinct branches, of which we interpret one as more demanding – the demanding landscape. This is in turn divided into two parts: The first of these we interpret as the immense power of nature, dangerous to challenge, and it consists of three distinct clusters.

#### Cluster Number One

The first cluster collects concepts that deal with an inhospitable and threatening nature. It is as if taken from a horror movie with veils of mist, dangerous marshes, dead trees, ravens, predators, bats, and spiders. Several classic fairy tales and myths, such as the Grimm’s fairy tales, take place in such environments where witches and trolls dwell. The birds found here, such as ravens and crows, are intelligent birds loaded with a fateful and desolate symbolism: the birds of Odin (Table 3). The dead standing tree is an international danger symbol for products that kill, like the fire and the skull. This infertile, barren landscape should be avoided. The only thing that grows here is death cap, everything else is dead. However, there is a fascinating attraction to approaching these environments. In the literature, such landscapes can be found in, for example, Dante’s Divine Comedia (Alighieri, 2018) and Tolkien’s Mordor, in The Lord of the Rings (Tolkien, 2007). In some parts of the world, extreme forms of these landscapes could be found, such as the Death Valley desert in California. Major natural disasters, such as volcanic eruptions and tsunamis, also lead to this kind of landscape, as well as extensive war. The closest we can think of in the Nordic region are areas affected by large forest fires.

**Table 3:** Variables regarding objects and phenomena in the natural environment in southern Sweden that are included in Cluster 1.

Variable number	Object
87	The dead standing tree
85	The big fallen tree
120	Death cap
67	Rain clouds
72	Cloudburst
66	The dramatic clouds
73	Misty vapor
74	Haze
10	Tarn
11	Marsh, swamp, and bog
124	Wolf
125	Bear
126	Predator (lynx, wolverine, brock, and fox)
133	Raven
134	Magpie
135	Crow/rook
129	Bat
161	Frog
55	Wasps
58	Ants/anthill
56	Bees
57	Beetles
59	Spiders/spider-webs

We associate the phenomenon with feelings of angst and escape, with a landscape where the god Odin – the god of wisdom and death – and his assembly of ravens (Huginn and Muninn) and wolves (Geri and Freki) rule (Ellis Davidson, 1990). Another association is the death kingdom of Hades: the realm of Hades is dark, misty, and gloomy. Here, the vast crowd of dead move like shadows in an eternal haze, impenetrable to the sun. It is a dark and dismal realm where bodiless ghosts flitter across gray fields. The Homeric poets found that all – heroes and villains alike – came to rest in the gloom of Hades. In the Homeric hymns, the realm is a dark, damp, and moldy place (Rayor, 2014). We propose calling the phenomenon this cluster points at “**the Landscape of Death**.”

#### Cluster Number Two

The second cluster contains extremes of weather phenomena, directly dangerous to human life and health such as hurricanes and floods after rain and storm. In this cluster, there is also the snake, an animal that many feel discomfort toward and fear of (Table 4). Terrible storms are central topics in many religions, such as in the Bible, with the flood of earth in the story of Noah’s Ark. Storms are also central themes in many modern books and movies.

**Table 4:** Variables regarding objects and phenomena in the natural environment in southern Sweden that are included in Cluster 2.

Variable number	Object
63	The storm/hurricane
81	The snowstorm
62	Strong gusts
71	The full day rain
70	The heavy rain
82	The hailstorm
64	The thunderstorm
75	The thick fog
60	Snakes

In many parts of the world, the snake is associated with thunder, storm, and lightning. In the Nordic Pre-Christian religion, the battle between the thunder god Thor and the malicious and enormous snake Jörmungandr takes place (Ellis Davidson, 1990). It is about the immense and sublime power of nature, which sometimes expresses itself as a leviathan fury of nature, where man is often small and defenseless.

We propose calling this phenomenon “the Leviathan Storm” or “**the Storm.**”

#### Cluster Number Three

The phenomenon we find in the third cluster is about seeking a natural landscape that is powerful and sublime, but where a human is capable of mastery. The temptation is in the wilderness, far from built-up areas, where you have some protection at the fire and a shelter. The fire stands for safety and warmth (Table 5). The adventure is characterized by concepts like rafts and ruins. It is about challenging and exploring high mountains, the sea and wild rapids. During the adventure, focus is on survival, while it is also possible to sometimes dream away, thinking, for example, at the campfire. Such challenges can be more or less dangerous.

**Table 5:** Variables regarding objects and phenomena in the natural environment in southern Sweden that are included in Cluster 3.

Variable number	Object
176	The campfire
262	The sound of a fire
175	The cave
174	The hut/cubbyhole
173	The ruin
172	The deserted house
179	The deserted wild garden
203	The climbing tree
24	The mountain
5	The cliff beach
233	The sail boat
235	The fishing boat
231	The canoe/kayak
230	The raft
232	The rowboat
14	The river
15	The rapid
255	The torrential stream
1	The wild sea
253	The roaring sound from the wild sea

The god Heimdallr was sent by the other gods to the people, to teach them how to survive in the wilderness. He taught them to tame the holy fire, and crafts so that they could build houses and boats. He was also a guardian, with the sharpest hearing and vision that one could imagine. With his horn, he sent important messages to the people, especially if dangers were approaching (Ellis Davidson, 1990).

This nature archetype describes adventurers and their landscape, such as explorers of the Himalayas and the North Pole. Thor Heyerdahl, who explored the oceans of the world, with the raft Kon-Tiki and the papyrus boat Ra, belong in this nature archetype. Authors who focus on this phenomenon are Jack London, Mark Twain, teenage book author Enid Blyton, and Daniel Defoe with Robinson Crusoe, which has inspired, among other things, the creation of the popular TV series “Survivor.” This nature archetype of course also attracts people to more moderate challenges, such as hikes with overnight stays in tents, mountain hikes, canoe trips, etc.

We suggest that we call this phenomenon “Adventurous Wilderness” or “**the Adventurer**.”

The second distinct branch in this cluster is about challenges in a more populated landscape. These two nature archetypes have two sides, which can be interpreted as divine twins (Ward, 1968). At first, they are appealing and can be experienced as restorative, at least in the short term. They are, however, also challenging, and can be frightening, even lethal.

#### Cluster Number Four

This landscape leads to thoughts of summer holidays with sun, swimming, and boat trips. In Sweden, sun and heat are often welcome, not least in spring when they chase away the winter cold. The Swedes’ summer vacation is often about finding sandy beaches to sunbathe and swim, or maybe taking a boat out to the archipelago.

The Palace or Manor house stands as a symbol of power, civilization, success, growth, and wealth. Here, the sun shines from a clear blue sky over the beach and a calm sea (Table 6). The sun is a symbol of prosperity, usually represented by an important god of different religions: in ancient Egypt by the god Ra; in ancient Greece by the god Apollo, and in the Shinto of the goddess Amaterasu, and also in ancient Sweden. One of the oldest mighty gods – perhaps the oldest – was Ullr: the glowing, brilliant, and bright god, the god of hunting and of the sun (Ellis Davidson, 1990). Through the centuries and until now, the sun has been associated with power and wealth, not least in recent years, with lots of literature, films, and TV series with rich, powerful people living in castle-like buildings in landscapes with beaches, plenty of sun, and large motor boats. The growth of civilization is also represented in the form of new roads and crowds of people. This nature archetype is for Swedes one of the most attractive. However, as it often involves crowding and traffic jams, it can lead to stress.

**Table 6:** Variables regarding objects and phenomena in the natural environment in southern Sweden that are included in Cluster 4.

Variable number	Object
166	The palace/mansion
52	Sunshine
53	Sunrise
54	Sunset
51	The clear blue sky
3	The sandy beach
68	The rainbow
61	The breeze
2	The calm sea
6	The archipelago
234	The motor boat
208	The barbed wire fence
164	Crowds of people
190	The new railroad
188	The new road

In the landscape with this kind of growth and development, everything is new, and nature becomes oppressed. Among farmers, too much rain as well as too much sun have been associated with concern: The sun of prosperity could unexpectedly change to a burning sun with drought and bad harvest as a consequence. In the Greek mythology, Icarus flew against the sun, despite his father’s warnings (Pinsent, 1982). The wax that held together the feathers in his suit melted by the heat from the sun, so Icarus fell to his death. The story is about hubris, that power and assets are blinding, so people refuse to listen to warnings. The barbed wire, walls and throng, can also tell an ancient story of defense of wealth. Today’s climate change, where even large rivers and lakes dry out, mean that people in these areas need to search for more fertile landscapes, but these people often meet walls and barbed wire. This is probably no new phenomenon, rather an ancient one. The cluster points to development that leads to a grand existence in the sun for some, others being excluded.

We propose to call this, “**the Landscape of the Sun**.”

#### Cluster Number Five

The fifth cluster points to an old-fashioned farmhouse in a forest landscape. Here, the farm is used in accordance with nature. People gather assets from forests and lakes to store berries, mushrooms, and fish. They live on farms with farm animals like sheep and cows. It is a winter landscape, with the moon and a night sky covered by stars.

The Nordic moon god Mani was enchantingly beautiful (Ellis Davidson, 1990). The moon is seductive and attractive but is simultaneously associated with evil powers, certain creatures, and death. In the Swedish mythological tradition, darkness and moonlight are associated with creatures such as trolls, gnomes, dwarfs, and ghosts. Two birds are present in the cluster: the swan and the pigeon, both representing purity, spirituality, and contacts with afterlife – and the cemetery is also a part of the cluster (Table 2). It is midwinter, a complete and devotional silence prevails. It is about the threats of winter coldness, darkness, and the unknown – to vigil until the morning light and safety – such as in the ancient Swedish Lucia celebrations (Hellquist, 1922; Alver, 1976).

Several poems and literature references are found in Sweden, in older hymns such as Silent night, and in Viktor Rydbergs “Tomten” which is about a Swedish folklore watchman, a household spirit which can be compared with the British brownie (Fredriksson, 2018).

“Tomten”

*Midwinter’s nightly frost is hard* –

Brightly the stars are beaming;

Fast asleep is the lonely Yard,

All, at midnight, are dreaming.

Clear is the moon, and the snow-drifts shine,

Glistening white, on fir and pine,

Covers on rooflets making.

None but Tomten is waking.

Gray, he stands by the byre-door,

Gray, in the snow appearing;

Looks, as ever he did before,

Up, at the moonlight peering;

Looks at the wood, where the pine and fir

Stand round the farm, and never stir;

Broods on an unavailing

*Riddle, forever failing*.

This old landscape, with tiny farms inside large, old coniferous forests is part of the Swedish folk soul, important in novels by Selma Lagerlöf, poetry by Gustaf Fröding and in stories for children by Elsa Beskow. This landscape, which both scares and attracts, is also found in many Nordic fairy tales, about trolls and elves and the like, but this kind of landscape also seems important in Western fairy tales such as “Hansel and Gretel” and “Snow White and the Seven Dwarfs.” Parts of this landscape also moves into Swedish cities, as folklore parks and museums, for example, Skansen in Stockholm.

This nature archetype, with winter, cold, large coniferous forest and simple rural life, both attracts and frightens. The darkness and the cold frighten, while the attraction lies in the activities and in the sense of staying in a landscape with large coniferous forests and lakes, which bear a distinct character of Swedish nature. Activities related to old-time farming, such as fishing and berry and mushroom picking, attract many Swedes. It is a landscape of melancholy, magical and at the same time bewitching and even eerie. We propose to call this “**the Landscape of the Moon**.”

### The Restorative Landscape

The restorative cluster has two branches. One consists of a more pastoral cluster of activities in natural environments. This cluster in turn divided into four separate clusters. The other cluster, that is alone, relates to pure rest and recovery.

#### Cluster Number Six

The sixth cluster points to a pastoral landscape one walks through, where you move through lush deciduous woods of different species and then walk through the open countryside, over plains, and hills, where small creeks and brooks run (Table 7).

**Table 7:** Variables regarding objects and phenomena in the natural environment in southern Sweden that are included in Cluster 6.

Variable number	Object
185	Nature trail with cairns
28	The rolling countryside
187	Old country road
189	Old railroad
184	The hiking route
29	The plain
26	The hill
27	The ridge
45	Oak forest
43	Beech forest
44	Birch forest
42	Alder forest
40	Mixed forest
41	Mixed deciduous forest
35	The spinney
36	The forest edge
89	Oak
96	Rowan
98	Bird cherry tree
92	Maple
95	Alder
93	Elm
94	Aspen
90	Lime-tree
91	Ash-tree
97	Birch
47	Timber stack
48	Branches on the ground
101	Shrubbery
46	Juniper forest
30	Heather moorland
4	Shore meadow
12	Brook
13	Creek

In Nordic mythology, Odin often travels on long walks in the landscape, to meet human beings and solve problems; disguised, often with gray cape and wide slouch hat. For his help, he often has his loyal friend Hœnir as a guide and pathfinder. A third person who usually accompanies is Loki. Odin and Loki are both seen as ambiguous and difficult to understand, where Loki is the god who is the most enigmatic and hardest to comprehend. He often gives good help, but deep down he is wicked and willing to betray (Ellis Davidson, 1990). In the Lord of the Rings, a similar theme is found with the three walkers Frodo, Sam, and Gollum. Frodo must solve a serious problem; Sam is the loyal helpful friend while Gollum is a deceitful companion. Possibly, this theme reflects difficult internal dialogues, which have characterized mankind for millennia, and which individuals have to contend with on long walks.

More than anything else, hiking is a bodily act that unites man with the landscape. You do not only observe the landscape as a view: you move through it, where all the senses are present. During a long walk you more or less could become absorbed by the landscape. It is an activity during which the embodied-self experiences the natural landscape. Long pilgrim walks often take place in beautiful, enchanting landscapes which are crucial factors in these walks, as it is through the embodied and sensory interactions with the surrounding nature that pilgrims can have personally meaningful experiences (Scriven, 2014). Known pilgrim paths are the Christian Camino in Spain, the Buddhist Shikoku and the Shinto Sangu in Japan, where the path, the landscape and the individual person become interwoven. The pilgrim has left his/her daily routines, and on these walks, in slow pace, the pilgrim lowers his/her guard, physically and mentally. This results in the pilgrim opening up to take in the surroundings, enabling meditation at a deeper level. On these paths, there are moments and meetings that facilitate an inner dialogue, whether understood to be with oneself or with the Divine (Maddrell et al., 2015).

Wandering is a recurring theme in many books and films, such as “Here is your life” by the Swedish Nobel Prize winner Eyvind Johnson and “Rasmus and the vagabond” by the children’s book author Astrid Lindgren. Hiking is a popular leisure activity, not least in Sweden, where nature trails are expanded throughout the country.

We propose to call this “the Landscape of the Wanderer” or “**the Path**.”

#### Cluster Number Seven

The seventh cluster is about sensory experiences; sight, hearing, and scent, often associated with the changes of the seasons and water: dripping water as the snow melts, leaf cracking, but also autumn leaves. Large parts of the cluster are about the power of life tied to water, and perceived by subtle, sensual experiences. Many variables are linked to wet areas such as lakes and beaches, reeds, and rushes (Table 8). On the other hand, the stone, which is found in this cluster, is stable and does not change character. Even the shifts of the seasons are in themselves eternal. It is about marveling about this cycle of life and the eternity. In the pre-Christian Sweden, there were the goddess of water and the creation of life; Nerthus (Vikstrand, 2001; Ingelman-Sundberg, 2004).

**Table 8:** Variables regarding objects and phenomena in the natural environment in southern Sweden that are included in Cluster 7.

Variable number	Object
49	The cycle of the seasons
249	The sounds of the succession of the seasons, such as water dripping from icicles
76	Dew
77	Frost
256	Rippling water
69	The silent rain
236	The scent after rain
250	The sounds of the wind
251	The sough of the wind in the trees
254	The murmur of waves
107	Leafing
239	Scent of deciduous forest
238	Scent of coniferous forest
54	Bumblebees
260	Buzz from bumblebees and bees
108	Autumn leaves
248	Scent of autumn
210	The plowed field
240	Scent of soil
110	Plants at the seashore such as lyme grass and seaweed
109	Plants at the lake shore such as cotton grass, sedges, and horsetail
242	Scent of lake
229	Fishnet
112	Reed
252	Sough in the bulrush
241	Scent of mire
121	Ferns
153	Dragonflies
17	The old rock
19	Bare bedrock, outcrop
18	Boulder
20	Big rocks
122	Moss
123	Lichens

The Japanese and Chinese gardens often contain rocks, moss, and water that can symbolize the theme. Swedish writers who have worked with the theme are Swedish Nobel Prize winner Harry Martinson, and Erik Axel Karlfeldt with the poem “Autumn’s Spring” and Karin Boye with the poem “Yes, of course it hurts.” It is about a phenomenon that treats the rhythm of life, the waves, and lapping of waves – the lapping of life. The old stone that does not change is in contrast to everything that changes.

We propose calling this “the Eternal Cycle,” or “**Eternity**.”

#### Cluster Number Eight

This is about an older culture and farming landscape where there are plenty of traces of human work and values, where parts of the landscape form central concepts in Swedish folk tradition and poems. Here you will find the “warden trees,” the grove, meadows, grazing lands, mountain farms, hay-fences, and several different types of fields (Table 9). The fields are enclosed with roundpole or dry-stone fences. In contrast to the landscape that is set forth in cluster 5, this landscape is bright and more associated with joy and expectations. Some old, larger deciduous trees in the cultural landscape were seen as holy in the ancient Nordic region – the warden trees. The god of sowing, harvesting, and fertility is Freyr (Ellis Davidson, 1990), and he belongs here.

**Table 9:** Variables regarding objects and phenomena in the natural environment in southern Sweden that are included in Cluster 8.

Variable number	Object
197	Grove
196	Pasture
214	Oat field
213	Barley field
218	Flax field
215	Rye field
216	Wheat field
222	Headland (field-edge)
223	“Field-holm” – a non-arable outcrop or small insular area inside a field
219	Hay meadow
220	Newly mown hay
221	Hay rack
205	Cairn
206	Dry-stone fence, low stonewall
207	Roundpole fence
195	Grazing pasture
186	Country lane (wheel track with green in the middle)
183	Forest path
171	Shieling
199	Utility garden (orchard and kitchen garden)
177	Warden tree
84	The unusual tree
86	The big, old tree

This landscape is the image of the Swedish cultural landscape, which has been described by Gustaf Fröding, Erik Axel Karlfeldt, August Strindberg with the People of Hemsö, and songs by Evert Taube. Important in this context are also Astrid Lindgren’s children’s characters, like the children in Noisy village, and Emil of Lönneberga.

We propose to call this “the Pastoral Cultural Landscape” or “**Fertility**.”

#### Cluster Number Nine

People have a positive attitude toward the animals that appear in cluster nine. Many of these animals live in or near cities, towns, and villages, such as starlings, swallows, larks, hedgehogs, roe deer, hares, and squirrels. Wild animals have played an important role in people’s everyday lives in the Nordic countries, not least the friendly or felicific animals (Table 10). In Nordic mythology, the whole world consists of a huge tree: Yggdrasil. For the tree and thus the world to last, it is monitored, among other things, by a number of guardian animals. At the top there are two birds of prey, an eagle “Hräsvelg,” and a hawk “Väderfölne,” as well as deer. A squirrel, Ratatoskr, runs with messages from the roots up to the eagle (Enoksen, 2006). In the Bible, the wild animals also have a significant role. Therefore, Noah saves the animals in the giant ark. In Nordic folklore, which is still alive in northern Sweden and Finland, some birds were seen as sacred: the eagle, the crane, the cuckoo, and especially many seabirds as they master several elements – air, land, and water. The most sacred was the swan, which could travel back and forth to the kingdom of death (in cluster 5). Small birds, such as the robin, the lark, and the blackbird, were considered to be soul-birds. They were manifestations of the deceased’s souls, ancestors, or guides/followers to the deceased (Salo, 2018). In fact, C.G. Jung emphasized the *animal archetype*. It is sublime, he said, and represents the divine side of the human psyche (Jung, 1953, 1992; Hannah, 2006). The elk is an animal that has had an important place in mythology and religion in the Nordic countries since time immemorial. Hedgehogs were considered to give luck to the home and hurting a hedgehog might bring great misfortune to the home (Schön, 2002). These animals could also be connected to fylgjur. In Norse mythology, a fylgja was an individual’s alter ego and guardian spirit, which sometimes could show itself in animal form. Good and kind people had nice fylgjur (Mundal, 1974).

**Table 10:** Variables regarding objects and phenomena in the natural environment in southern Sweden that are included in Cluster 9.

Variable number	Object
127	Large mammals, like roe deer and elk
128	Small mammals like squirrel, hare, and hedgehog
258	The sound of animals
137	Small birds
257	Rustle from small birds and animals
146	Chaffinch
140	Lark
141	Nightingale
145	House sparrow
139	Swallows
143	Wagtail
138	Blackbird
144	Starling
147	Winter birds like bullfinch and great tit
132	Woodpecker
148	Crane
149	Ducks
142	Cuckoo
130	Birds of prey
131	Owl

Even today, several of these animals have a clear symbolic meaning. In fables or proverb, animals may represent certain roles or human characters, where they express clear human qualities, such as the wise owl, the happy lark, the elk as the king of the forest, etc. Walt Disney has this theme in movies, such as Snow White, where birds, hares, and deer help out. There is also an interest in having animals in the gardens or near the house. Today you often find animals of stone or metal, adorning gardens at people’s homes.

We suggest that this cluster be called “Likable Animals,” the “Guardian Animals,” or “**the Guardian**.”

#### Cluster Number Ten

Here are several objects clustered, that in the Nordic region are associated with summer, holidays, enjoyment, relaxation, and vacations, such as water lilies, butterflies, birdsong, flowers, fruit trees, arbors, and hammocks (Table 11). Here is also the bridge, the small harbor, and the scent of sea – something that relates to holiday life on the coast, which is loved by Swedes. The cluster contains no wildlife but is human-made or influenced by humans. There is nothing that threatens the idyll; no job and no duties.

**Table 11:** Variables regarding objects and phenomena in the natural environment in southern Sweden that are included in Cluster 10.

Variable number	Object
178	Fruit trees
202	Arbor
180	The pond
111	Water lilies
152	Butterflies
83	Greenery
194	Lea
245	Scent of grass
247	The fragrance of spring
102	Spring flowers such as wood anemone and blue anemone
103	Early summer flowers such as lily of the valley and primrose
104	Midsummer flowers such as poppy, marguerite, and corn flower
105	Late summer flowers such as tansy and willow herb
106	Forest blossoms such as arctic starflower, wood sorrel, and twinflower
244	The fragrancy of flowers
201	The garden of enjoyment and delight, with hammock…
259	Bird song
151	Bird’s nest/bird house
181	Bird table
182	Bird bath
192	Tree avenue
193	Line of pollarded willows
224	Bridge
227	Channel
228	Boathouse
236	The little harbor
225	Jetty
243	Scent of sea

In the Nordic pre-Christian religion, you could hear about the god Balder’s dwelling Breidablick, located by a river, and mentioned as “A more beauteous place may not be found.” It is clean, green, and indeed most pleasant. Other references are the fruit tree of the Garden of Eden, the epicureans’ pleasure, the flowers of the hedonists. It is about relish, rest, and fertility, to find the way to the soul’s enjoyment. Additional sources are Bellman: “Sit down around the spring here”; Astrid Lindgren:” Seacrow Island,” and ballads by Evert Taube and, more contemporary, Ulf Lundell.

We propose to call this “**the Garden of Eden**.”

## Discussion

More and more, nature is described in forms of archetypes in e.g., landscape planning, landscape architecture, and geography (e.g., Hartel et al., 2018; Nogué and Wilbrand, 2018, Evers et al., 2019). In Landscape and Memory, Schama (1995) focuses on the relationship between human perception of the physical environment, not least the natural environment and landscape, and human traditional culture. These relationships are intertwined with each other and preserved in legends. Schama finds evidence of this in different cultures, in different eras, all over the world. The myths are revealed in ceremonies, poems, and visual arts, and Schama refers to certain natural elements as archetypes. In this article, we have investigated whether people group different types of phenomena in nature into categories that can be described as archetypes. We asked 547 people in southern Sweden to comment on 257 different natural phenomena. These were grouped into 10 distinct clusters, which we interpreted as archetypes that can be related to powerful old southern Scandinavian myths.

The result as a whole shows clear phenomena that we judge as providing relevant information. Tables 2–11 show that respondents were thorough when filling in the form. For example, in cluster 3, we find that two questions that are far apart in the questionnaire cluster together strongly (176: “the sound of a fire” and 252: “the campfire”). There are several other examples. These show a validity in the material. We find that the clusters are reasonable; hardly any variables have ended up in the wrong cluster. The questionnaire puts sounds in nature and smells in nature separately. Nevertheless, campfires and the sound of the fire came to cluster together very strongly, which indicates that respondents filled out the forms carefully. Some parts could be expected, such as that several species of birds and species of trees, respectively, should end up in the same cluster. However, it is interesting that ravens end up among large predators and spiders in cluster 1, while small birds end up with squirrels and hares in cluster 9. Another evidence that the result is valid is that the 10 clusters describe characteristic features of Sweden’s landscape. For instance: cluster 5 can be interpreted as the taiga, the world’s largest land biome, which covers a large part of northern Sweden. Cluster 6 describes the biome that makes up a large part of southern Sweden: the nemoral temperate broadleaf forest.

The 10 clusters can be attributed to two main types: the first five are more demanding while the five later ones are restorative. Clusters 1–3 are about dangers in nature, where the first two deal with environments that are life-threatening and the third is about a demanding and challenging wilderness, where you will encounter hard fascination while there is also scope for mindfulness and soft fascination – you can, for example, drift off into dreams when you rest by the campfire (Kaplan and Kaplan, 1989). Clusters 4 and 5 relate to civilization, where the modern society in cluster four and the darkness and cold of winter in cluster five are demanding. Clusters 6–9 refer to a restorative, pastoral landscape, while cluster 10 is a landscape of pure rest and enjoyment.

We suggest that these archetypes have a strength and vitality that can both be perceived and communicated and can affect the visitors’ psychophysiological status.

### On Archetypes and Innate Preferences Obtained Through Evolution

Initially, the article described how many researchers called for useful descriptions of qualities in green areas. Some researchers suggest that archetypal descriptions of landscapes might work because these provide a holistic, fair description of natural areas (Hreško et al., 2015; Cullum et al., 2017). Others also make connections to Jung’s theories about archetypes, and moreover to environmental psychology (Bourassa, 1988; Nash, 1997; Wilkinson, 2015; Xing and Chen, 2018). Thus, they can be used to explain how nature areas can function health-promoting.

Over the past 35 years, more and more research has shown that staying in special natural environments is curative for humans, with many research results pointing to the fact that staying in nature provides a recovery from high levels of stress (Egorov et al., 2016; Nilsson et al., 2019). In these cases, nature is described as restorative. Restoration is defined as the recovery of diminished daily functions and capabilities, largely during people’s free time (Han, 2018). Restorative environments are defined according to von Lindern et al. (2016) as environments that both permit and promote restoration. In parallel, there is evidence showing that people’s ability to process crises increases when they stay in natural areas. In these cases, nature is described as instorative. The word instorative relates to instauration – meaning an act of instituting or establishing something. Instorative effects are about how nature areas appear to act as catalysts; necessary in accelerating the processing of crises so that reorientation is achieved faster (Hartig et al., 1996; Stigsdotter and Grahn, 2002, 2003; Nilsson et al., 2019; Dushkova and Ignatieva, 2020).

In terms of both the restorative and instorative potential of nature, reference is made to man’s long history of functioning and survival in nature, about evolution and the archaic man. People who suffer from high levels of stress restore their capacities when staying in natural environments but not so in built-up environments (Ulrich, 1993; van den Bosch and Bird, 2018). When grief, depression and life crises take over, people seek clear relationships that can provide consolation and offer solutions. People can be difficult to relate to, as well as cities and settlements. Natural areas, however, seem to be able to offer consolation and reflections on the life situation that can work instorative; develop coping strategies and lead to ways out of crises (Grahn et al., 2010; Sahlin et al., 2012; Pálsdóttir et al., 2014). However, most of the studies – especially regarding restorative effects – have used extreme types of natural environments and built-up environments: tranquil alluring and bright natural environments against the gray noisy city. Though, it is unlikely that all natural environments will reduce people’s high stress levels (Bixler and Floyd, 1997). It is, therefore, important to be able to describe the qualities of the outdoor environments concisely, with a limited number of factors in order not getting lost in the details.

Studies dealing with the development of health-promoting green spaces often refer to the qualities that Appleton (1975) emphasizes in his Prospect-Refuge theory (e.g., Senoglu et al., 2018). Other authors refer to Orians (1986) and his savannah theory (e.g., Townsend and Barton, 2018) or to Wilson (1984) and his biophilia theory (e.g., Browning et al., 2014). These theories highlight individual aspects in natural environments. Kaplan and Kaplan’s (1989) Attention Restoration Theory contains four qualities which have been used to develop green areas (e.g., Stack and Shultis, 2013; Jang and Son, 2020). The Perceived sensory dimensions (PSDs) developed since the 1980s (van den Bosch et al., 2018; Stigsdotter et al., 2020), contain the qualities mentioned in the theories above (Appleton, 1975; Wilson, 1984; Orians, 1986; Kaplan and Kaplan, 1989) as well as some additional. The PSDs address the general content of green areas based on aspects such as species richness, space, seclusion, or tranquility. These are of a general nature and work all over the world, both in cities and in the countryside; for example, in Malaysia (Mansor et al., 2017), Serbia (Vujcic and Tomicevic-Dubljevic, 2018), China (Chen et al., 2019), Canada (Lockwood, 2017), Iran (Memari et al., 2017), and Denmark (Plambech et al., 2015). We suggest that these descriptions need to be supplemented with depictions of archetypal content, which include plant and animal species, watercourses, landscape formations, seasons, and symbolic significance. We assume that the archetypal content of nature influences visitors’ emotions and psychophysiological reactions; that the nature archetypes affect the stress system as well as the anti-stress system. The stress system includes the HPA axis which regulates cortisol levels, and the sympathetic nervous system. The anti-stress or the oxytocinergic system involves the parasympathetic nervous system and is linked to stimulation of social interaction, decreased stress levels and stimulation of restorative processes and growth (Uvnäs-Moberg, 2013).

According to Jung, archetypes have guided man through crises during all times (Jung, 1968; Knox, 2004). As stated by Jung, the archaic man had a close relationship to nature as well as to the gods who revealed themselves in natural environments in the form of the sun, the thunder, and other powerful natural phenomena (Sabini, 2008). Nature archetypes are supposed to be innate and are assumed to guide people regarding typical social phenomena and patterns of action that have followed human beings through evolution. The most widely accepted interpretation today of Jung’s archetypes, is that they are innate dispositions to detect and react on objects or coherent patterns, so called archetypal images or symbols (de Coro, 2018). “The archetype is a tendency to form (…) representations of a motif – representations that can vary a great deal in detail without losing their basic pattern” (Jung, 1964). Since nature has followed humans for millions of years through evolution, patterns of action in relation to typical characteristics and phenomena in nature should have been inherited. However, researchers so far have focused on how visits in natural areas have affected the sympathetic nervous system, and not realized the possibility of including the calm and connection system in their explanatory models (Grahn et al., 2010; Uvnäs-Moberg, 2013; van den Bosch and Bird, 2018). In a follow-up article, we intend to develop a model for how nature archetypes interact with the calm and connection system.

This study suggests that humans can discover certain archetypes in nature. The study has been conducted entirely in a Southern Scandinavian context and, therefore, needs to be repeated in other parts of the world. For example, it would be interesting to see how many of the proposed 10 nature archetypes that will be re-discovered in other studies. Such could be interpreted to be more basic, innate while others could be interpreted to be more culturally bound.

## Data Availability Statement

The raw data supporting the conclusions of this article will be made available by the authors, without undue reservation.

## Ethics Statement

The studies involving human participants were reviewed and approved by The Swedish Ethical Review Authority, Lund, Sweden. The patients/participants provided their written informed consent to participate in this study.

## Author Contributions

JO and PG developed the idea for the study together, worked together to create the questionnaire and interpreted the results and took responsibility for the overall structure and writing of the final manuscript. PG was responsible for materials and methods. JO contacted the subjects and collected all material. All authors contributed to the article and approved the submitted version.

### Conflict of Interest

The authors declare that the research was conducted in the absence of any commercial or financial relationships that could be construed as a potential conflict of interest.
